# Age is not a limiting factor for brachytherapy for carcinoma of the node negative oral tongue in patients aged eighty or older

**DOI:** 10.1186/1748-717X-5-116

**Published:** 2010-12-09

**Authors:** Hideya Yamazaki, Ken Yoshida, Tadayuki Kotsuma, Yasuo Yoshioka, Masahiko Koizumi, Souhei Furukawa, Naoya Kakimoto, Kimishige Shimizutani, Tsunehiko Nishimura

**Affiliations:** 1 Department of Radiology, Kyoto Prefectural University of Medicine, 465 Kajiicho Kawaramachi Hirokoji, Kamigyo-ku, Kyoto 602-8566 Japan; 2 Department of Radiology, National Hospital Organization, Osaka National Hospital, Hoenzaka 2-1-14 Chuo-ku, Osaka city, Osaka 540-0006 Japan; 3 Department of Radiation Oncology, Osaka University Graduate School of Medicine, Yamadaoka 2-2, Suita, 565-0871 Osaka, Japan; 4 Department of Maxillo-Facial Radiology, Osaka University Graduate School of Densitry, Yamadaoka 1-8, Suita, 565-0871 Osaka, Japan

## Abstract

**Background:**

To examine the role of brachytherapy for aged patients 80 or more in the trend of rapidly increasing number.

**Methods:**

We examined the outcomes for elderly patients with node negative oral tongue cancer (T1-3N0M0) treated with brachytherapy. The 21 patients (2 T1, 14 T2, and 5 T3 cases) ranged in age from 80 to 89 years (median 81), and their cancer was pathologically confirmed. All patients underwent definitive radiation therapy, with low dose rate (LDR) Ra-226 brachytherapy (n = 4; median 70Gy), with Ir-192 (n = 12; 70Gy), with Au-198 (n = 1) or with high dose rate (HDR) Ir-192 brachytherapy (n = 4; 60 Gy). Eight patients also underwent external radiotherapy (median 30 Gy). The period of observation ranged from 13 months to 14 years (median 2.5 years). We selected 226 population matched younger counterpart from our medical chart.

**Results:**

Definitive radiation therapy was completed for all 21 patients (100%), and acute grade 2-3 mucositis related to the therapy was tolerable. Local control (initial complete response) was attained in 19 of 21 patients (90%). The 2-year and 5-year local control rates were 91%, (100% for T1, 83% for T2 and 80% for T3 tumors after 2 years). These figures was not inferior to that of younger counterpart (82% at 5-year, n.s.). The cause-specific survival rate was 83% and the regional control rate 84% at the 2-years follow-up. However, 12 patients died because of intercurrent diseases or senility, resulting in overall survival rates of 55% at 2 years and 34% at 5 years.

**Conclusion:**

Age is not a limiting factor for brachytherapy for appropriately selected elderly patients, and brachytherapy achieved good local control with acceptable morbidity.

## Background

Oral tongue carcinoma is a highly curable cancer when treated with radiation therapy, especially interstitial brachytherapy [1]. Iridium-192 (Ir-192) hairpins or cesium-137(Cs-137) needles are usually used for low-dose-rate (LDR) interstitial radiotherapy in Japan. We used a high-dose-rate (HDR) remote-controlled after-loading system, using an Ir-192 microsource, the MicroSelectron-HDR (Nucletron, Veenendaal, The Netherlands) installed in 1991. Since with this system there is no risk of radiation exposure except to the patient, HDR makes it possible to treat patients in a normal ward, so that the quality of life during treatment may be better. We have already reported on the outcome of HDR brachytherapy for early oral tongue cancer which included a prospective Phase III study [2-4]. In addition, we reported that the efficacy of brachytherapy for T3 oral tongue cancer, especially when using HDR, was enhanced by its excellent dose distribution [5]. The number of elderly patients in Japan has been increasing steadily because of advances in both health and medical care and the leading cause of death among the elderly is cancer. The number of people aged 80 or over reached 7,130,000 in Japan in 2007, which counts for more than 5% of the population. The problems involved in treating older patients with cancer are time pressing [6]. As aging is a highly individualized process, the indication, strategy, and techniques of radiation therapy for the elderly should not be defined exclusively by chronologic landmarks [6]. We studied 21 80-year-old or older patients with oral tongue cancer treated by brachytherapy. Since to the best of our knowledge, there have been no previous reports regarding such patients, we conducted this retrospective review of the feasibility of brachytherapy for elderly patient with T1-3N0 oral tongue cancer.

## Methods

### Patients

Between 1967 and 2004, 21 patients (9 males and 12 females) with previously untreated mobile tongue cancer were treated with radiotherapy at Osaka University Hospital and Osaka National Hospital. Patients treated with radiotherapy combined with chemotherapy were excluded from the study. All tumors were histologically identified as squamous cell carcinoma. Table 1 lists patient and treatment characteristics. The patients' median age was 81, ranging from 80 to 89. There were 2 T1, 14 T2, and 5 T3 tumors (UICC TNM classification of 1987). During the study period, we also treated about 700 patients with T1-3N0 oral tongue carcinoma [4], with the elderly group accounting for 3% of all patients. The age of the 21 patients ranged from 80 to 89 years (median 81) at the start of radiation therapy, and the male-to-female ratio was 9:12. Performance status (PS) was classified as 0-1, based on the World Health Organization classification. For this study, the clinical records of consecutive these 21 patients from our database were reviewed (Table 1). To compare the result of treatment to younger counterpart, we reviewed population adjusted (sex, T-stage, with external radiotherapy) 226 patients treated during same time period. The background comparison was shown in Table 2.

**Table 1:** Characteristics and clinical background of the study population

**No**.	Sex	Age	T	Longest diameter	Short diameter	Thickness	Tumor type	External RT dose	Brachytherapy dose	Radioactive Source	Initial response	Survival	Disease control	Outcome	Complications	Comorbidity
		(year)		(mm)	(mm)	(mm)		(Gy)	(Gy)			(year)		(status)		
1	F	86	2	25	15	5	exo		73	Ir	CR	1.2	NED	DID		
2	F	85	2	25	13	5	sup	30	60	Ra	CR	7	NED	DID		
3	M	82	1	20	11	5	sup		70	Ra	CR	2	NED	DID (senile decay)		
4	F	82	1	20	7	3	exo+ulc		65	Ir	CR	6	NED	DID (senile decay)		Hypertention
5	M	82	2	35	23	15	ins+ulc	30	60	Ir	CR	3	NED	DID		
6	M	82	2	25	12	10	NA		68	Ir	CR	1	NED	Alive		TIA
7	F	81	2	25	12	10	ulc		76	Ir	CR	14	nodal failure 4M	Alive (salvaged by surgery)		
8	F	82	2	38	17	10	ind		48	ms	CR	2.5	NED	Alive	Ulcer and bone exposure after biopsy	
9	M	81	2	24	15	12	ind		60	Ir	CR	1.4	nodal failure 8M	Dead (nodal failure)		
10	F	80	2	23	7	5	ind		70	Ir	CR	12	NED	DID (senile decay)		Hypertention
11	M	80	2	23	18	7	exo		75	Ir	CR	6	NED	Alive		Hypertention
12	M	80	2	NA	NA	NA	NA		70	Ir	CR	2	NED	DID		
13	M	80	3	42	25	25	ulc	33	60	ms	CR	1.4	NED	DID		
14	M	80	3	42	23	13	ulc+ind	30	85	Ra	CR	3	NED	DID (senile decay)		
15	F	83	3	42	20	20	ulc+ind	30	70	Ra	PR	1	nodal failure 0M	Dead (nodal failure and local failure)		
16	F	89	3	50	30	20	ulc+ind	36	32	ms	CR	1.3	NED	DID		
17	F	83	2	31	24	8	ind+exo		70	Ir	CR	4	NED	DID		
18	M	81	3	35	28	15	ulc+ind	36	54	Ir	CR	0.9	NED	DID		
19	F	80	2	25	20	10	ind		70	Au	PR	1.8	local failure 4M	Dead (local failure)		
20	F	80	2	25	20	6	ind	50	54	Ir	CR	2	NED	Alive		
21	F	82	2	27	22	12	exo		54	ms	CR	2	NED	Alive	Mild ulcer, pain	

Ir; Ir-192, Ra; Ra-226, ms; Ir-192 microSelectron-HDR, Au; Au-198 grain

sup; superficial type, exo;exophytic type, ind; indurative type, ulc; ulcerative type

DID; died for intercurrent disease, DT; death for tongue cancer, NED; no evidence of disease, NA; not avairalble

TIA; transient ischemic attack

**Table 2:** Background of aged patients and younger conterpart

				Aged (80-)		Control (-79)	
				(N = 21)		(N = 226)	
Age	Age	Median (Range)		81 (80-89)		56 (18-79)	
Gender	Male			9	(43%)	101	(45%)
	Female			12	(57%)	125	(55%)
T classification	T1			2	(10%)	30	(13%)
	T2			14	(67%)	146	(65%)
	T3			5	(24%)	50	(22%)
	Long diameter	(mm)		30 ± 8		26 ± 9	
	Short diameter	(mm)		18 ± 7		18 ± 8	
	Thickness	(mm)		11 ± 6		9 ± 6	
Source	Ra-226			4	(19%)	72	(32%)
	Ir-192			12	(71%)	120	(63%)
	Au-198			1	(5%)	0	(0%)
	MS-HDR			4	(19%)	34	(15%)
External radiotherapy	Brachytherapy only			13	(80%)	165	(73%)
	Combined with external radiotherapy			8	(20%)	61	(27%)
Prescribed dose	Brachytherapy	Median (Range)(Gy)	LDR	70 (54-85)		70 (50-112)	
			HDR	54 (32-60)		60 (42-60)	
	External radiotherapy	Median (Range)(Gy)		LDR		30 (12-60)	

HDR; high dose rate, LDR; low dose rate

### Radiation therapy

All implantation was done under local anesthesia. For patients in the LDR group, the treatment sources consisted of an Ir-192 pin for 12 patients, a Ra-226 needle for 4 and a ^198^Au grain for one patient. Each needle was implanted with the Paterson-Parker system using a reference point 5 mm distant from the implant plane. The median dose and range for the LDR group treated with brachytherapy only was 70 Gy (61-84 Gy). Patients in the HDR group received a total dose of 60 Gy in ten fractions during one week at 5 mm distance from the radioactive source. Two fractions were administered per day. The time interval between fractions was more than 6 hours. Dose rates at the reference points for the LDR group were 0.30 to 0.8 Gy/h, and for the HDR group 1.0 to 3.4 Gy/min. Patients were followed up for at least 13 months or until their death, with a median follow-up time of 2.5 years (range: 1.3 - 14 years). Large T2 tumor or more including ulceration or thicker tumor received external irradiation. A total of 8 patients (T2: 3, T3: 5) underwent external radiotherapy using a Co-60 teletherapy unit or a linear accelerator. These patients received 2-3 Gy per fraction for a median dose of 30 Gy (30 - 50 Gy), and were treated with a single lateral field that involved the primary site and the upper jugular lymph nodes. Nutrition support was given by nasal tube feeding during brachytherapy. No patient required tracheostomy. The routine follow-up interval was 1 month for the first year, two months for the second year, and 3 - 6 months thereafter. We examined the outcomes in terms of local control, lymph node control, cause-specific and overall survival. Early toxicities were assessed by Common Toxicity Criteria version 3 (CTC v3). Late toxicities were counted if soft tissue (ulceration lasting 3 months or more) and/or bone (bone exposure and necrosis) reactions occurred.

### Statistical Analysis

For a statistical analysis, a Student's t-test for normally distributed data and the Mann Whitney U-test for skewed data were used. The percentage was analyzed using a Chi-square test. Local control and survival data were estimated according to the Kaplan-Meier method, and were examined for significance with a logrank test. All analyses used the conventional p < 0.05 levels of significance.

## Results

### Local control, regional control, cause-specific and overall survival

**Figure 1:**
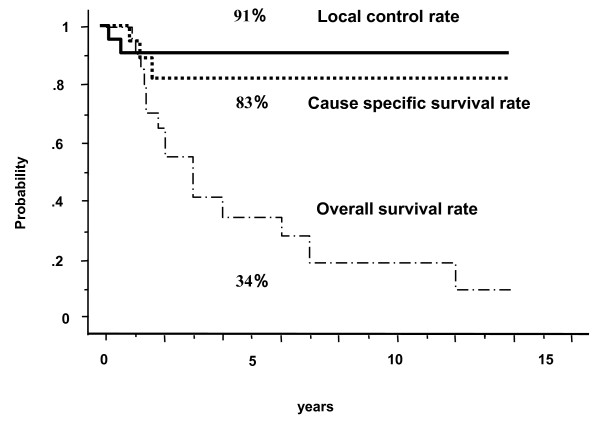
**Local control, cause specific survival and overall survival rates for patients 80 or more with oral tongue cancer treated with interstitial radiotherapy**. solid line; local control rate, dotted line; cause specific survival rate, dashed line; overall survival rate.

**Figure 2:**
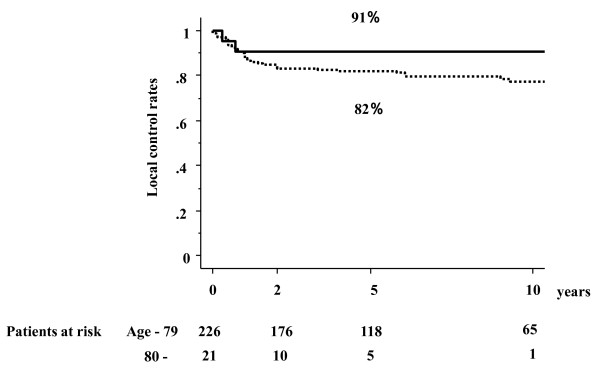
**Local control rates according to age**. Aged patients 80 or more showed no inferior local control rate to younger counterpart.

The 2- and 5-year local control rates for the 21 elderly patients were both 91% (Figure 1). The 2-year (5-year) local control rates for T1, T2, T3 tumors were 100% (100%), 83%, (83%) and 80% (not available), respectively (n.s.). These figures was not inferior to that of younger counterpart (82% at 5-year, Figure 2 n.s.). Two patients showed local recurrence. An 83-year-old female (ID15) received external radiotherapy for lymph node metastasis found just after completion of brachytherapy, but local recurrence appeared and resulted in death. One more local failure occurred in an 80-year-old female with T2N0 oral tongue cancer (ID 19) treated with the Ir-192 source. During the first night of treatment in the RI ward, she tried to brush her teeth and pulled out the guide gutter of the Ir-192, so that the Ir-192 needles were replaced with Au-198, resulting in partial response and recurrence 4 months later. The 2-year and 5-year cause-specific survival (CSS) rates were both 83% (83% and 78% in control group), but the respective overall survival rates were 55% and 34% (83% and 76% in control group). Incidence of lymph-node metastasis was 21% at 2 years (34% in control group) and all four recurerce appeared at ipsilateral side. Of the 4 patients who showed nodal failure, three underwent surgery, one of whom could be salvaged. Actuarially 12 patients died because of intercurrent disease or old age. The follow-up for 5 patients had to be terminated because the patients or their family requests.

### Tolerance and Complications

Grade 2-3 acute mucositis, pharyngitis for combined external radiotherapy and oral mucositis for solely brachytherapy, occurred but were acceptable. No grade-4 skin or mucosal acute reactions were documented. The intensity of acute reactions in the elderly patients was almost the same as that observed in younger patients. Late reactions after brachytherapy comprised one bone exposure and/or two ulcer formations lasting 3 months more (2/21 = 10%; Table 1). One case showed tongue deformation with ulceration scar. In previous cohort [4], 10 to 30% of delayed reaction was found according to treatment volume and addition of external radiotherapy. Aged patients showed similar ratio of delayed reaction.

## Discussion

The patients 80 years old or older among those who were treated by brachytherapy accounted for about 3% of our cohort. The incidence of carcinogenesis among this age group is currently unavailable, but oncologists are treating increasing numbers of elderly cancer patients, so that we should be more deeply concerned about treatment strategies for these patients. The deterioration of biological functions associated with aging leads to a diminished reserve capacity and increased vulnerability to age-related diseases and overall forces of mortality [6-8]. As the effects of aging depend on the individual, they manifest themselves with great variability and heterogeneity, thus making it extremely difficult if not impossible to determine a standard therapy for elderly patients based only on chronologic landmarks. When deciding on a personalized mode of treatment for older patients, it is important to assess each patient's quality of life and life expectancy. Prognostic factors related to the tumor (TNM stage, pathology, etc.), physical and/or psychological status (PS, etc.), and social support should be taken into account when estimating the outcome of treatment and life expectancy of elderly patients. However, the major part of prospective trials is carried out with patients younger than 70 so that little evidence regarding elderly patients is available.

Generally, local treatment is more appropriate than systemic therapy for the elderly. Standard chemotherapy, especially combination treatment, is not encouraged because of elderly patients' physiologically impaired functions and diminished reserve capacity of important organs [9-11]. Unsatisfactory outcomes of combination therapy have been reported [8], although better results with less toxic antineoplastic agents or reduced doses of chemotherapeutic agents especially designed for elderly patients with non-Hodgkin's lymphoma have been reported [12]. Moreover, the rates of acute adverse effects, morbidity, and mortality remain high for the elderly, so that extended radical surgery is not encouraged for the same reasons. It is important for their quality of life and life expectancy to attain local control of symptomatic primary lesions. Carefully planned radiation therapy for the elderly is expected to become increasingly important [13]. A prospective study has also reported the usefulness of radiotherapy for esophageal cancer in elderly patients [14], and found that patients with good PS could tolerate doses that administered according to a standard radiotherapy schedule [9]. Our findings agreed with this study in that the completion rate of radiotherapy and local control rate for elderly patients were not inferior to those for younger patients.

One of the limitations of this study is that its retrospective nature leads to a lack of detailed information about co-morbidity. This is important because cardiovascular and pulmonary diseases as well as diabetes and other diseases are more pronounced in elderly than younger patients. In addition, as mentioned in results, unexpected accidents will occur more frequently in elderly than younger patients. We found four cases of hypertention and a TIA records in patients' charts, however, they were able to be diagnosed as candidates for brachytherapy with local anesthesia and we noted that adverse reactions such as mucositis in HDR brachytherapy were similar for elderly patients: spotted mucositis started to appear three days after the end of brachytherapy while confluent mucositis developed and reached a peak at ten days, but disappeared by the fourth to eighth week without any major complications [2]. Fortunately, we did not encounter the aspiration pneumonia after brachytherapy in current study. Severe deterioration in QOL, such as speech disturbance, swallowing function loss, and frequent short hospital stay were also not a case enhanced than younger counterpart. Although the number of patients in this series was too small to draw definite conclusions regarding efficacy, late toxicity and tolerance, our data suggest the potential benefits of brachytherapy for elderly patients.

Because radiation therapy is considered to be a minimally invasive treatment procedure, it has the advantage of preserving the shape and functions of the tongue. Brachytherapy was historically performed with Ra-226, which involved exposure of the surrounding tissue. To minimize undesirable radiation to normal tissues, an afterloading technique using Ir-192 was implemented. This LDR brachytherapy has been widely used since and become the gold standard in brachytherapy. Many institutes have reported successful results for tongue cancer treated with LDR brachytherapy [2,15]. Since then, HDR brachytherapy using a remote afterloading technique has been introduced in several brachytherapy centers, including ours [2-4]. We previously reported our phase III data and a retrospective review with good results for T1-3 N0 patients to show the comparable outcome of HDR. However, retrospective reviews including ours reported that older patients aged 65 or over showed poorer local control than their younger counterparts [3,4]. In a 648-patient cohort, 5-year local control rates were 87% for T1, 78% for T2, and 68% for T3 in younger patients, but 72% for T1, 67% for T2, and 54% for T3 in elderly patients aged 65 or over (p < 0.05) [4]. These findings prompted us to examine the background characteristics of older patients. We found that one possible explanation for poor local control was poor oral hygiene including dental factors in the elderly in previous study [12], which could be modified by careful intervention. In addition, in the study reported here, we found that patients aged 80 or over showed good outcome including four locally controlled HDR patients. Therefore age is not a sole factor on a local control rate by brachytherapy, other confounding factor such as tumor, oral hygiene, PS, co-morbidities have affected outcomes. Although further studies are needed to establish optimum schedules and techniques, elderly patients with good PS may tolerate brachytherapy schedules so that the advisability of definitive radiation therapy should be considered.

In conclusion, patients aged 80 or over showed results comparable to those for their younger counterparts, and an aggressive approach for appropriately selected elderly patients achieved good local control.

## Competing interests

The authors declare that they have no competing interests.

## Authors' contributions

HY conceived of this study and drafted manuscript. KY participated in the design of this study. TK and YY participated in the statistical analysis. MK, SF, NK, KS and TN participated in its design and coordination and helped to draft the manuscript. All authors read and approved the final manuscript.
